# Supplementary material for: The orphan nuclear receptor Nurr1 is responsive to non-steroidal anti-inflammatory drugs
Source: Commun Chem. 2020 Jul 3;3:85. doi: 10.1038/s42004-020-0331-0 (PMC9814838; doi:10.1038/s42004-020-0331-0)
Supplement: Supplementary file 1 — Supplementary Information [file 42004_2020_331_MOESM1_ESM.pdf]

The orphan nuclear receptor Nurr1 is responsive to non-steroidal anti-inflammatory drugs

Sabine Willems[1], Whitney Kilu[1], Xiaomin Ni[1,2], Apirat Chaikuad[1,2], Stefan Knapp[1,2], Jan Heering[3], Daniel Merk[1]

[1] Institute of Pharmaceutical Chemistry, Goethe University Frankfurt, Max-von-Laue-Str. 9, 60438 Frankfurt, Germany

[2] Structural Genomics Consortium, BMLS, Goethe-University Frankfurt, 60438 Frankfurt, Germany

[3] Fraunhofer Institute for Molecular Biology and Applied Ecology IME, Branch for Translational Medicine and Pharmacology TMP, Theodor-Stern-Kai 7, 60596 Frankfurt, Germany

- Supplementary Information -

Table of contents

Supplementary Figures and Tables 2

Supplementary References 5

Supplementary Figures and Tables

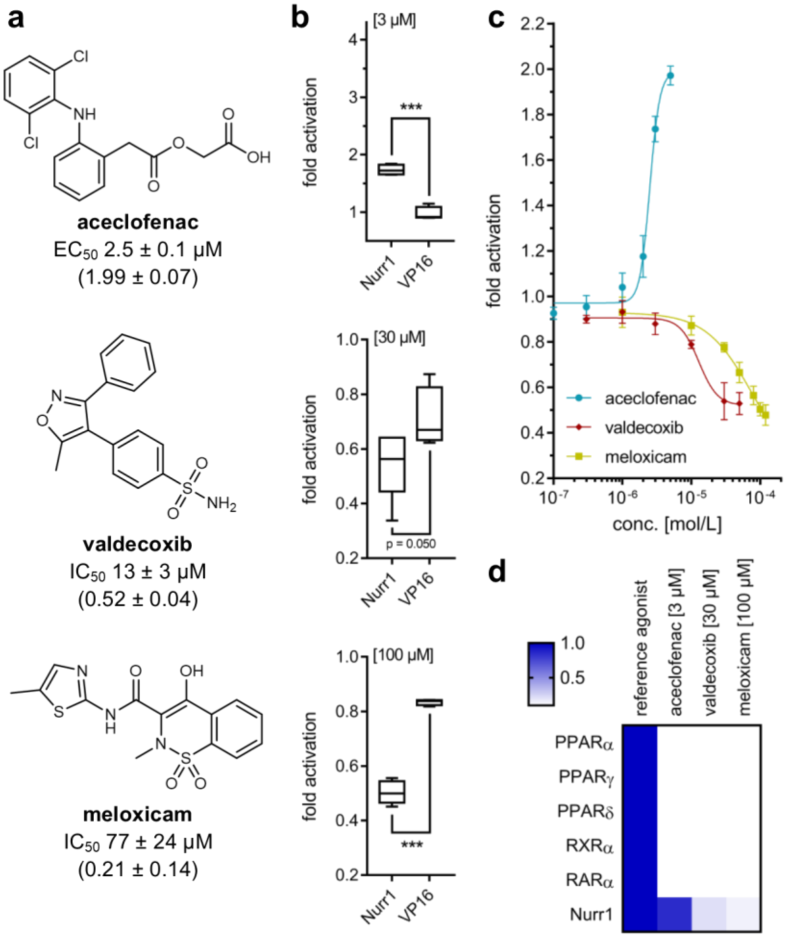

Supplementary Figure 1. Bidirectional modulation of Nurr1 activity by drug approved COX inhibitors. (a) Molecular structures and activities of Nurr1 modulators aceclofenac, valdecoxib and meloxicam. EC50 and IC50 values were determined in the Gal4-Nurr1 hybrid reporter gene assay and are the mean ± SD; n ≥ 3. (b) Control experiments employing a Gal4-VP16 hybrid receptor confirmed Nurr1 mediated activity of aceclofenac, valdecoxib and oxaprozin. Boxplots show: center line, median; box limits, upper and lower quartiles; whiskers, min/max; n ≥ 4. *** p < 0.001. (c) Gal4-hybrid reporter gene assay demonstrated Nurr1 activation by aceclofenac as well as inverse Nurr1 agonism for valdecoxib and meloxicam. Results are mean ± S.E.M.; n ≥ 3. (d) Selectivity profile of Nurr1 modulators over lipid activated transcription factors. Heatmap shows mean rel. activation which refers to reference agonists at 1 µM for PPARs (α: GW7647; γ: rosiglitazone; δ: L165,041), RXRα (bexaroten), RARα (tretinoin) and 100 µM for Nurr1 (AQ); n ≥ 4.

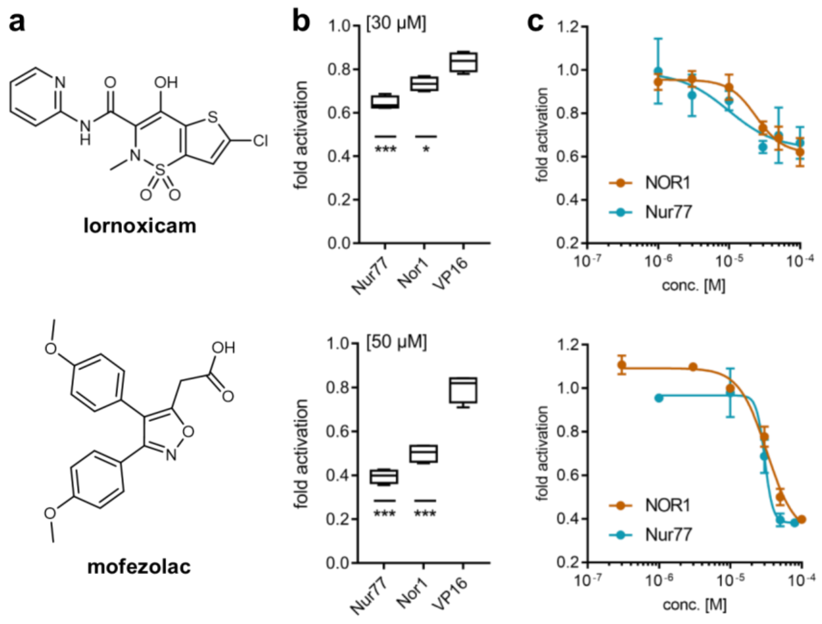

Supplementary Figure 2. Inverse agonists of related NR4A receptors Nur77 and NOR1 not affecting Nurr1 activity. (a) Molecular structures of NR4A receptor modulators lornoxicam and mofezolac. (b) Control experiments employing a Gal4-VP16 hybrid receptor confirmed Nur77 and NOR1 mediated activity of lornoxicam and mofezolac. Boxplots show: center line, median; box limits, upper and lower quartiles; whiskers, min/max; n ≥ 4. * $p < 0.05$, ** $p < 0.01$ *** $p < 0.001$. (c) Dose-response curves demonstrate dose-dependent inverse Nur77 and NOR1 agonism. Data are mean ± SD, n ≥ 3.

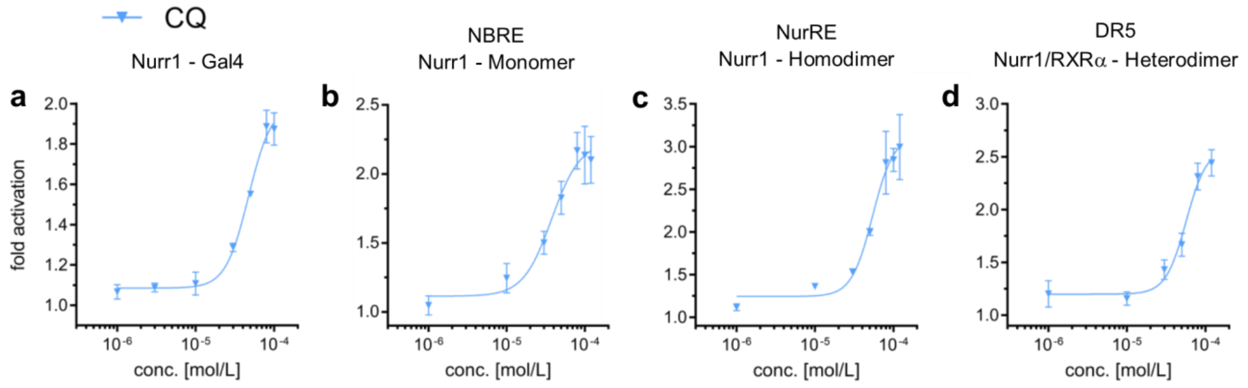

Supplementary Figure 3. Cellular profiling of Nurr1 modulator chloroquine (CQ). Data shown here are identical with Fig. 3a-d for CQ in the manuscript but y-axis scaling has been adapted here to depict the CQ dose-response. The Nurr1 activation efficacy of CQ is markedly lower compared to AQ. (a) Gal4-hybrid reporter gene assay demonstrated Nurr1 activation by CQ. (b-d) Nurr1 full-length reporter gene assays with the human Nurr1 response elements NBRE (Nurr1 monomer, b), NurRE (Nurr1 homodimer, c), and DR5 (Nurr1:RXR heterodimer, d) confirmed agonism of CQ. All cellular experiments were performed in transiently transfected HEK293T cells. Results are the mean ± S.E.M.; n ≥ 3.

Supplementary Table 1. Activity of NSAIDs on NR4A nuclear receptors Nur77 (NR4A1), Nurr1 (NR4A2) and NOR1 (NR4A3) determined in uniform Gal4-hybrid reporter gene assays in transiently transfected HEK293T cells. Activity was verified using Gal4-VP16[1,2] as control and only compounds with statistically significant ($p < 0.05$) activity on the respective NR4A receptor versus VP16 control are reported as active. EC_{50}/IC_{50} values are reported in [µM]. Values in parentheses are min./max. activation compared to 0.1% DMSO serving as vehicle. Data are the mean ± SD, n≥3.

	**Nur77**	**Nurr1**	**NOR1**
**meclofenamic acid**	EC_{50} 3.9 ± 0.7 (3.3 ± 0.4)	EC_{50} 4.7 ± 0.1 (3.52 ± 0.05)	EC_{50} 7.9 ± 0.8 (5.5 ± 0.6)
**meloxicam**	IC_{50} 73 ± 2 (0.23 ± 0.02)	IC_{50} 77 ± 24 (0.2 ± 0.1)	IC_{50} 84 ± 17 (0.1 ± 0.1)
**lornoxicam**	IC_{50} 9.4 ± 6.3 (0.63 ± 0.09)	-	IC_{50} 24 ± 3 (0.62 ± 0.02)
**aceclofenac**	-	EC_{50} 2.5 ± 0.1 (1.99 ± 0.07)	-
**mofezolac**	IC_{50} 30 ± 1 (0.38 ± 0.02)	-	IC_{50} 33 ± 5 (0.32 ± 0.10)
**oxaprozin**	IC_{50} 16 ± 5 (0.2 ± 0.1)	IC_{50} 40 ± 6 (0.26 ± 0.08)	IC_{50} 22 ± 4 (≥ 0.00)
**valdecoxib**	-	IC_{50} 13 ± 3 (0.52 ± 0.04)	-
**parecoxib**	IC_{50} 23.7 ± 0.2 (0.1 ± 0.0)	IC_{50} 13.4 ± 0.3 (0.48 ± 0.01)	IC_{50} 25 ± 4 (0.22 ± 0.05)

Supplementary References

1. Sadowski, I., Ma, J., Triezenberg, S. & Ptashne, M. GAL4-VP16 is an unusually potent transcriptional activator. *Nature* **335**, 563–564 (1988).

2. Budzyński, M. A., Puustinen, M. C., Joutsen, J. & Sistonen, L. Uncoupling Stress-Inducible Phosphorylation of Heat Shock Factor 1 from Its Activation. *Mol. Cell. Biol.* **35**, 2530–2540 (2015).
